# Supplementary figures and images for: The epidemiology of superficial Streptococcal A (impetigo and pharyngitis) infections in Australia: A systematic review
Source: PLoS One. 2023 Nov 30;18(11):e0288016. doi: 10.1371/journal.pone.0288016 (PMC10688633; doi:10.1371/journal.pone.0288016)

## Supporting Information 2

### Consideration of bias: funnel plots

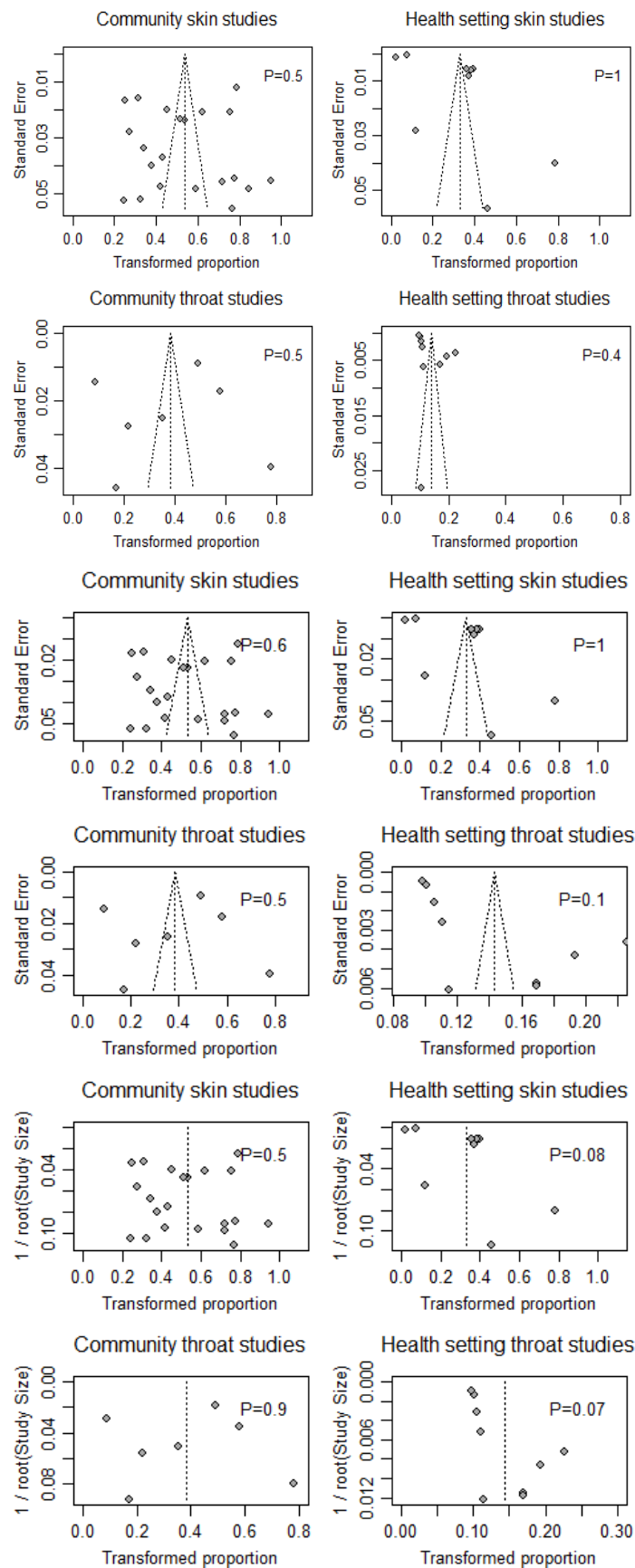

Supplement: S1 Fig — (PDF) [file pone.0288016.s001.pdf]
