# Supplementary material for: The epidemiology of superficial Streptococcal A (impetigo and pharyngitis) infections in Australia: A systematic review
Source: PLoS One. 2023 Nov 30;18(11):e0288016. doi: 10.1371/journal.pone.0288016 (PMC10688633; doi:10.1371/journal.pone.0288016)
Supplement: S1 Table — (PDF) [file pone.0288016.s002.pdf]

Supporting information 1
Summary of methods and baseline population characteristics of included articles.

Author	Title	Year of publication	Quality &	Age of population (yr)	%Aboriginal Torres Strait Islander	Location#	Study setting	Collection method	Person making diagnosis	Definition of skin disease	Definition of throat disease
Abdalla et al.	Hospital admissions for skin infections among Western Australian children and adolescents from 1996 to 2012	2017	Good	<16	6.7%	WA, State-wide	Inpatient of a hospital	Identification of inpatient diagnosis codes 'Pyoderma' and 'Impetigo'	Unclear (from medical record entry)	Impetigo and pyoderma as principal diagnosis for admission	-
Andrews et al.	A regional initiative to reduce skin infections amongst aboriginal children living in remote communities of the Northern Territory, Australia	2009	Good	<15	100.0%	NT, Arnhem land	School, GP practice and community	Screening of children within study setting	Doctor, nurse, community health officer	Pyoderma	-
Britt et al.	General practice activity in Australia 2001-02	2002	Good	All ages	1.3%	AUS	GP practice	Presentation for health consultation	Doctor	-	Tonsillitis
Britt et al.	A decade of Australian general practice activity 2003-04 to 2012-13: General practice series no. 34	2013	Good	All ages	1.3%	AUS	GP practice	Presentation for health consultation	Doctor	-	Tonsillitis
Carapetis et al.	Skin sores in Aboriginal children	1995	Good	< 18	100.0%	NT, Top End	School	Random selection of school children	Doctor, community health officer	Pyoderma	-
Carapetis et al.	Success of a scabies control program in an Australian Aboriginal community	1997	Fair to good	All ages	100.0%	NT, Minjilang	Community	Random selection of school children	Community health officer or medical student overseen by a doctor	Pyoderma, skin sores	-
Charles et al.	Trends in childhood illness and treatment in Australian general practice, 1971-2001	2004	Good	< 15	Not stated	AUS	GP practice	Presentation for health consultation	Doctor	-	Tonsillitis
Clucas et al.	Disease burden and health-care clinic attendances for young children in remote Aboriginal communities of northern Australia.	2008	Fair to good	< 5	100.0%	NT, East Arnhem	Community health clinic	Presentation for health consultation	Workers of Primary Health Care centres	Skin sores	Throat infection

Comino et al.	The general health of a cohort of aboriginal children (0-7 years) in Sydney	2017	Fair	>2, < 8	100.0%	NSW, Sydney	Home visits	Unclear selection process	Parental report	-	Tonsillitis
Danchin et al.	Burden of acute sore throat and group A streptococcal pharyngitis in school-aged children and their families in Australia	2007	Good	All ages	Not stated	VIC, Melbourne	GP practice	Random selection of families presenting to GP practice with a child 3-12 years of age	Doctor	-	Sore throat
Del Mar et al.	Incidence patterns of respiratory illness in Queensland estimated from sentinel general practice	1995	Fair to good	All ages	Not stated	QLD, statewide	GP practice	Cases reported in those presenting for GP appointment	Doctor	-	Sore throat: acute infection limited to tonsillitis and pharyngitis
Dossetor et al.	Pediatric hospital admissions in Indigenous children: A population-based study in remote Australia	2017	Fair to good	< 8	95.3%	WA, Kimberley, Fitzroy Crossing	Inpatient of hospital	Presentation to hospital for health consultation in a pre-defined cohort	Health professionals	Impetigo, skin sore	Tonsillitis/ pharyngitis
Heath et al.	Nutrition status of primary school children in Townsville	2005	Fair to good	>5, <10	45.9%	QLD, Townsville	School	Screening of school children	Healthcare worker	Skin sores	-
Hendrickx et al.	Ascertaining infectious disease burden through primary care clinic attendance among young Aboriginal children living in four remote communities in Western Australia	2018	Good	< 6	100.0%	WA, Western Desert	GP practice	Presentation for health consultation	Nurse, doctor	Skin sores	Throat infections
Heyworth et al.	Prevalence of non-specific health symptoms in South Australia	2001	Fair	> 15	Not stated	SA, State-wide	Home visit	Survey or randomly selected households	Self-report	-	Sore throat
Howarth et al.	Strongyloidiasis, Scabies and Impetigo–Household Clustering In A Northern Territory Community	2016	Good	All ages	100.0%	NT, remote Aboriginal community 550km from Darwin	Community	Presentation for health consultation	Health professional	Impetigo	-

Hoy et al.	A health profile of adults in a Northern Territory Aboriginal community, with an emphasis on preventable morbidities	1997	Fair to good	>20y	100.0%	NT, Coastal Aboriginal community	Community	Volunteers for health screening	Unclear	Skin sore	-
Ingarfield et al.	Acute upper respiratory infections in Western Australian emergency departments, 2000-2003	2011	Fair to good	All ages	Not stated	WA, Perth	Emergency department	Presentation to hospital for health consultation	Hospital worker	-	Acute pharyngitis/ acute tonsillitis
Kearns et al.	Clinic Attendances during the First 12 Months of Life for Aboriginal Children in Five Remote Communities of Northern Australia	2013	Fair to good	< 1	100.0%	NT, East Arnhem	Community clinic	Presentation for health consultation in pre-defined cohort	Primary Health Care clinic workers	Skin sores	Throat infection
Lai-Kwon et al.	Which dermatological conditions present to an emergency department in Australia?	2014	Fair	> 15	Not stated	VIC, Melbourne	Emergency department	Presentation to hospital for health consultation	Hospital worder	Impetigo	-
Leach et al.	General health, otitis media, nasopharyngeal carriage and middle ear microbiology in Northern Territory Aboriginal children vaccinated during consecutive periods of 10-valent or 13-valent pneumococcal conjugate vaccines	2016	Fair to good	< 7	100.0%	WA, NT, Top End	Community	All children within a community	Ear health research nurse	Impetigo	-
Lehmann et al.	Benefits of swimming pools in two remote Aboriginal communities in Western Australia: intervention study	2003	Fair	< 17	100.0%	WA, remote community 1000km from Perth	Community	Screen of all children in the community	Aboriginal healthcare worker, doctor, nurse	Pyoderma	-
Mackerras et al.	Growth and morbidity in children in the Aboriginal Birth Cohort Study: the urban–remote differential	2003	Fair to good	> 5, <15	100.0%	NT, Darwin	Community	Screening of a predefined birth cohort	Paediatrician	Infected skin sore	-
McDonald et al.	Apparent contrasting rates of pharyngitis and pyoderma in regions where rheumatic heart disease is highly prevalent	2007	Fair	All ages	100.0%	NT, remote community 80km from Alice Springs	Community	Screening of all households in a pre-defined location	Aboriginal research officers and researchers	-	Sore throat
McDonald et al.	Low rates of streptococcal pharyngitis and high rates of pyoderma in Australian	2006	Fair to good	All ages	100.0%	NT, Top End	Community	Households with known cases of ARF/ RHD	Researchers	Pyoderma	Sore throat

	aboriginal communities where acute rheumatic fever is hyperendemic										
McMeniman et al.	Skin disease in the first two years of life in Aboriginal children in East Arnhem Land	2011	Fair to good	< 2	100.0%	NT, East Arnhem Land	Community, community clinic	Presentation for health consultation	Community health Clinic workers	Impetigo	-
Morgan et al.	Problems managed by Australian general practice trainees: results from the ReCEnT (Registrar Clinical Encounters in Training) study	2015	Good	All ages	Not stated	AUS	GP practice	Presentation for health consultation	Doctor	-	Tonsillitis
Nimmo et al.	Group A streptococcal infection in an aboriginal community	1992	Fair to good	> 2, < 13	100.0%	QLD, Lockhart River community	Community	Screening of school children	Unclear	Impetigo	Pharyngitis-non-purulent
Pollard et al.	The prevalence of wheezing and other respiratory symptoms in schoolchildren aged six to eleven years from Perth, Western Australia	1971	Fair to good	> 6, < 11	Not stated	WA, Perth	School	Systematic sampling from school roll	Parental report	-	Sore throat or tonsillitis
SA dept. of Health and Aging report (2011)	Evaluation of the sustainability and benefits of swimming pools in the Anangu Pitjantjatjara Yankunytjatjara Lands (APY Lands) in South Australia : final report	2011	Fair to good	< 18	100%	SA, APY lands	School, community	Selection of children from the school roll	Doctor	Pyoderma	-
Streeton et al.	An epidemic of acute post-streptococcal glomerulonephritis among Aboriginal children	1995	Good	> 2, < 15	100.0%	QLD, remote communities 450km from Cairns	Community	Screening in community with APSGN epidemic	Unclear	Skin sores	-
Sullivan et al.	Swim for life Nauiyu - Aquatic recreation project	2008	Fair	>5, < 18	100.0%	NT, Daly River, Nauiyu Nambiyu	Community	Screening of school children	Community health clinic workers	Skin sores	-
Tasani et al.	The Importance of Scabies Coinfection in the Treatment Considerations for Impetigo	2016	Good	< 14	100.0%	NT, Top End and central	School, community, home visit	Screening of children in study setting	Research nurses	Skin sore requiring treatment	-

Telethon Kid's Institute	The Swimming Pool Study 2000-2006	2006	Fair to good	< 17	100.0%	WA, Pilbara and Gascoyne, Burringurrah and Jigalong	Community	Screening of children in the community	Paediatrician	Skin sore	-
Tong et al.	Trimethoprim-sulfamethoxazole compared with benzathine penicillin for treatment of impetigo in Aboriginal children: A pilot randomised controlled trial	2010	Fair to good	> 2m, < 17	100.0%	NT, Top End	Community	Screening of children in the community	Study investigators	Crusted or purulent impetigo	-
Van Buynder et al.	Streptococcal infection and renal disease markers in Australian aboriginal children	1992	Good	> 4, < 18	100.0%	NT, remote communities: two coastal, one desert	School	Random selection from the school roll	Unclear	Impetigo	-
Wong et al.	Outcome of an interventional program for scabies in an Indigenous community	2001	Good	< 6	100.0%	NT, large Aboriginal community	Community	Screening of children in the community	Doctor	Non-scabies pyoderma	-
Yeoh et al.	Are scabies and impetigo "normalised"? A cross-sectional comparative study of hospitalised children in northern Australia assessing clinical recognition and treatment of skin infections	2017	Good	< 16	74.1%	WA, Kimberley and Pilbara	Inpatient of hospital	Patients admitted to hospital	Prospective: paediatric clinician, retrospective: unclear (from medical records)	Impetigo	-

[#] Location abbreviations: AUS - Australia-wide, NT - Northern Territory, WA- Western Australia, QLD - Queensland, VIC - Victoria, SA - South Australia
Other abbreviations: Acute Post-Streptococcal Glomerulonephritis (APSGN)
[&] Overall quality of study from assessment with the Joanna Briggs Institute critical appraisal checklist for prevalence studies
