# Supplementary material for: The epidemiology of superficial Streptococcal A (impetigo and pharyngitis) infections in Australia: A systematic review
Source: PLoS One. 2023 Nov 30;18(11):e0288016. doi: 10.1371/journal.pone.0288016 (PMC10688633; doi:10.1371/journal.pone.0288016)
Supplement: Attachment — Submitted filename: Rebuttal for reviewers .docx [file pone.0288016.s006.docx]

Rebuttal from minor edits

Thank you for your feedback and suggestions on this work. The points offered for change will improve and clarify the research we are presenting here. We will break down our response to be comment specific.

Reviewer 1

1) We agree that it would be important to further clarify inclusion and exclusion criteria early. An extended inclusion and exclusion criteria has been added to lines 116- 122.
2) The aim of the systematic review is to document the epidemiology of the clinical presentations of probable sGAS infections in Australia. Our current practice is to treat these infections based on clinical presentation without taking microbiological samples or relying on these results to guide treatment. Therefore, by adhering to best practice, there will be no microbiological confirmation of sGAS infection and a systematic review focused on microbiologically confirmed sGAS infection would likely under-represent the overall burden of disease. A systematic review reporting prevalence of clinical presentations has greater clinical application and meaningful contribution to the current literature.

We agree that this needs to be clarified in the paper. Current explanations have been made line 91-2, 409-12. We have expanded on this point in line 93-95 and reflected on this limitation in lines 408-11.

3) Thank you for this feedback. We agree that placing our systematic review in a global context may be useful to clinicians and researchers.

We have addressed this 471-3 in the discussion.

Minor comments

- Point regarding current throat sGAS research has been clarified in line 95
- Line 229 – use of ‘bar’ here meaning ‘except for’. Happy to change to ‘but’ for clarity.

Reviewer 2

1) The screening process has been clarified in line 141-2.
